# HET0016 decreases lung metastasis from breast cancer in immune-competent mouse model

**DOI:** 10.1371/journal.pone.0178830

**Published:** 2017-06-13

**Authors:** Thaiz F. Borin, Adarsh Shankar, Kartik Angara, Mohammad H. Rashid, Meenu Jain, Asm Iskander, Roxan Ara, Iryna Lebedyeva, Hasan Korkaya, Bhagelu R. Achyut, Ali S. Arbab

**Affiliations:** 1 Georgia Cancer Center, Augusta University, Augusta, GA, United States of America; 2 Department of Chemistry and Physics, Augusta University, Augusta, GA, United States of America; University of Alabama at Birmingham, UNITED STATES

## Abstract

Distant metastasis is the primary cause of death in the majority of the cancer types. Recently, much importance has been given to tumor microenvironment (TME) in the development of invasive malignant tumors, as well as the metastasis potential. The ability of tumor cells to modulate TME and to escape immune-mediated attack by releasing immunosuppressive cytokines has become a hallmark of breast cancer. Our study shows the effect of IV formulation of HET0016 (HPßCD-HET0016) a selective inhibitor of 20-HETE synthesis, administered intravenously in immune-competent in vivo mouse model of murine breast cancer. 4T1 luciferase positive cells were implanted to the mammary fat pad in Balb/c mice. Treatment started on day 15, and was administered for 5 days a week for 3 weeks. The development of metastasis was detected via optical imaging. Blood, spleen, lungs, bone marrow and tumor were collected for flow cytometry, to investigate changes in myeloid-derived suppressive cells (MDSCs) populations and endothelial phenotype. Tumor and lungs were collected for protein analysis. Our results show that HPßCD-HET0016: (1) decreased tumor volume and lung metastasis compared to the vehicle group; (2) reduced migration and invasion of tumor cells and levels of metalloproteinases in the lungs of animals treated with HPßCD-HET0016 via PI3K/AKT pathway; and (3) decreased expression of pro-inflammatory cytokines, growth factors and granulocytic MDSCs population in the lung microenvironment in treated animals. Thus, HPßCD-HET0016 showed potential in treating lung metastasis in a preclinical mouse model and needs further investigations on TME.

## Introduction

A major challenge in treatment of breast cancer is its propensity for distant metastasis to the lung, brain, bone and liver [1–3]. Distant metastasis is the primary cause of death in majority of the breast cancer types and its dissemination represents poor prognosis of survival compared to non-metastatic diagnoses [4]. Indeed, only 6% of patients with breast cancer are diagnosed with metastatic disease at the time of initial presentation, however 30% of all patient with breast cancer will eventually progress to metastatic disease [5].

Metastasis involves distinct steps that require detachment of cancer cells from the primary tumor, development of an aggressive phenotype, subsequent survival in transit, cooption on stroma of distant organs, formation of micro metastases, and finally full colonization [6].

The 'seed-and soil' hypothesis has been postulated based on the interactions between tumor cells and the corresponding microenvironment, wherein the tumor microenvironment (TME) is created by reciprocal influences between malignant cells, non-transformed cells, and their surrounding extracellular matrix. Evidently, intercommunication between cells is steered by tangled and dynamic network of cytokines, chemokines, growth factors, inflammatory and matrix remodeling enzymes against a background of major perturbations to the physical and chemical properties of the tissue [7].

In our previous studies, we have observed that 20-Hydroxyeicosatetraenoic acid (20-HETE), an active product of arachidonic acid metabolism by cytochrome P450, can activate several intracellular protein kinases including extracellular signal–regulated kinases (ERK) 1/2, phosphatidylinositol-3-kinases (PI3K) and protein kinase B (AKT), and growth factors such as vascular endothelial growth factor (VEGF) or receptors such as epidermal growth factor receptor (EGFR) leading to increased proliferation and neovascularization in U251 glioma cells and MDA-BM-231 metastatic breast cancer cells [8–10]. 20-HETE also can stimulate the production of various pro-inflammatory mediators such as prostaglandin E2 (PGE2), tumor necrosis factor alpha (TNF-α), and chemokines such as interleukin (IL)-8, IL-12, IL-14 [11].

Our ongoing investigations and recently published reports indicated that IV formulation of HET0016 with 2-Hydroxypropyl Beta Cyclodextrin (HPßCD) (HPßCD-HET0016), a 20-HETE synthesis inhibitor, not only decreased the tumor size but also inhibited the invasiveness of these cancer cells in animal models of human glioma [9]. This novel observation caught our attention, which led us to postulate whether HPßCD-HET0016 could be a potential therapeutic drug to control distant metastasis.

The purposes of this study were to determine the effect of HPßCD-HET0016 in controlling both primary and distal metastasis in a syngeneic breast cancel model developed by 4T1 breast cancer cells in immunocompetent Balb/c mice. Our data showed that HPßCD-HET0016 treatment decreased not only the primary tumor size but also distant lung metastasis. The effects are due to decrease in growth factors such as EGF and VEGF; pro-inflammatory chemokines and interleukins such as stromal cell-derived factor 1 alpha (SDF-1α), IL-17A, IL-1B and IL-4; matrix metalloproteinases (MMPs) such as MMP-2 and -9, and granulocytic myeloid-derived suppressor cells (G-MDSCs) population in lung microenvironment in treated animals.

## Materials and methods

### Ethics statement

All of the animals used in the experimental procedures were approved by the Institutional Animal Care and Use Committee and Institutional Review Board of Augusta University (2014–0625). All animals were kept under pathogen-free conditions at room temperature (21 to 25°C) with exposure to light for 12 hours and 12 hours in the dark. Food and water were offered *ad libitum*. Body weight was measured twice weekly as an indicator of overall animal health. All animal experiments were performed according to the NIH guidelines under ketamine/xylazine intraperitoneal or isoflurane inhalational anesthesia. All efforts were made to ameliorate the suffering of the animals during euthanasia using CO_2_ overdose followed by cervical dislocation.

### Cell line

4T1 murine breast cancer cell line was originally obtained from the American Type Tissue Culture Collection (ATCC), and modified by Dr. Hassan Korkaya (Augusta University) to express the luciferase gene reporter. The cell line was grown in Roswell Park Memorial Institute 1640 medium (RPMI) (Thermo Scientific), supplemented with 10% fetal bovine serum (FBS) (Nalgene-GIBCO), 2mM glutamine (GIBCO, Grand Island, NY, USA) and 100U/ml penicillin and streptomycin (GIBCO, Grand Island, NY, USA). MDA-MB-231 human breast cancer cell line was also used in this study, obtained from ATCC. The cell line was grown in Dulbecco’s modified Eagle’s medium (DMEM) high glucose (4.5 g/L) (GIBCO, Grand Island, NY, USA) supplemented with 10% FBS, 2 mM L-glutamine and 100U/ml penicillin and streptomycin. Both cell lines were cultured in a humidified chamber (Thermo Scientific) with 5% CO_2_ and at 37°C. As soon as these cells reached 80% confluence were harvested and seeded for *in vitro* study and 4T1 cells re-suspended in RPMI serum free media for tumor implantation.

### Migration and invasion assay

Wound healing assay was performed to detect the potential of HPßCD-HET0016 treatment to decrease migration malignant cells. 4T1 luciferase positive cells and MDA-MB-231 cells achieving 80–90% of confluency in 6 well plates were starved overnight with 0.5% FBS for cell cycle synchronization. Then, cells were treated with 100 μM of HPßCD-HET0016 for 24 and 48 hours in 2% FBS media, and microphotographed every 24 hours. The wound size was measured using Image J software (NIH) by drawing a rectangular region of interest to quantify the visible area of wound.

The invasiveness of breast cancer cells was tested in 24 trans-well plates with 8 μm inserts in PET track-etched membranes (Corning, Inc.) coated with 25 μg of matrigel matrix. 2.5 x 10^4^ cells/insert in serum free media were added into the upper compartment of the chamber with or without 100 μM of HPßCD-HET0016, while 750 μL of 10% FBS completed media was added to the lower compartment. For negative and positive controls, media with 0.5% and 10% FBS were used, respectively.

After 48 hours insert membranes were washed, fixed and stained with 0.05% crystal violet to detect the migrated/invaded cells. The counting was made with an inverted microscope (Nikon Eclipse E200, Melville, NY, USA) and the invasion rate was calculated by dividing the average number of treated cells that migrated and invaded the matrigel membrane per area by the average number of the positive control cells that did so, as described by Borin et al. [12].

### Establishing lung metastasis in breast cancer mouse model

Eighteen female Balb/c mice (Jackson Laboratory, Main USA), between 4–5 weeks of age and weighing 16-18g, were used for this experiment. 50,000 4T1 luciferase positive cells were cultured, prepared in matrigel (50 μL per mouse), and implanted into the 4^th^ mammary fat pad of the mice with the help of Dr. Korkaya, under anesthesia using intraperitoneal injection of Xylazin/Ketamine mix at a dose of 10 mg/kg, 100 mg/kg body weight, respectively.

In vivo optical images were obtained every week to keep track of metastasis development by injecting 100 μL of luciferin (3 mg/mL) intraperitoneally followed by acquiring bioluminescence signal for 60 sec. with spectral AmiX optical imaging system (Spectral instruments imaging, Inc. Tucson, Az), and the photon intensity/mm/sec was determined by Amiview software (version 1.6.0). The animals were anesthetized using an isoflurane vaporizer chamber (2.5% Iso: 2–3 L/min O_2_) and maintained under anesthesia during the procedure. Body weight of each animal was recorded every 3^rd^ day, until the end of the study to monitor well-being of the animals. The animals were divided into two groups, vehicle and HPßCD-HET0016 treated. The vehicle group (n = 9) received 30% of 2-Hydroxypropyl Beta Cyclodextrin (HPßCD—Sigma-Aldrich, St. Louis, MO), a complex to obtain water soluble IV formulation of HPßCD-HET0016, and treatment group (n = 9) received 10 mg/kg/day of HPßCD-HET0016 intravenously by tail vein, (dissolved in DMSO and 30% HPßCD, made fresh every day before administration), as reported in our previous study [10]. Treatments were administered 5 days a week, for 3 weeks, starting from day 15 of tumor implantation. The animals were euthanized at the end of week 6. Blood, spleen, lungs, bone marrow and tumor were collected for additional analysis.

### Immunohistochemistry staining

After animals’ perfusion, the primary tumor tissues were collected, kept in 3% paraformaldehyde and 3% sucrose solution, processed and paraffinized. Lung sections were stained for hematoxylin and eosin (H&E) to detect clear metastatic foci. Tissue sections were also stained for metastatic common markers such as E-cadherin (1:100—Bioss), N-cadherin (1:100—BD Biosciences), MMP-9 (1:100—Bioss), MMP-2 (1:200—Neomarkers), CD44 (1:100—Abcam) and CD24 (1:100—Santa Cruz). In brief, the sections were de-paraffinized, retrieved by boiling, incubated with protein block followed by primary antibody diluted in PBS, and kept at 4°C overnight. Then, the sections were washed with PBS and incubated with the secondary antibody and HRP Polymer Quanto (Ultravision Quanto Detection system HRP kit, Thermo Scientific) as recommended by the suppliers. Then, the sections were rinsed with PBS, incubated with diaminobenzidine tetrachloride (DAB) substrate, counterstained with hematoxylin, dehydrated, and coverslipped.

For analysis, five randomly selected areas were photographed at 40x magnification from each section and quantified the number of labeled and unlabeled cells per area determined by two independent observers using Image J software (NIH).

### Western blot analysis

Lungs (n = 4 vehicle and n = 4 treated group) were collected, snap frozen, and processed in tissue RIPA buffer with protease and phosphatase cocktail inhibitor (Thermo Scientific), to extract protein. Once proteins were extracted, it was quantified using Pierce BCA Protein Assay Kit (Thermo Scientific), loaded, and probed to identify the targets of HPßCD-HET0016 in metastasis inhibition; such as: MMP-9 (1:1000 –Abcam), MMP-2 (1:1000 –Neomarkers), N-cadherin (1:1000—BD Biosciences), E-cadherin (1:500—Bioss), pAKT, total AKT, pNFκB (p65 subunit), total NFκB (p65 subunit), pERK1/2 (p44/42 MAPK) and total ERK1/2 (p44/42 MAPK) (1:1000—Cell Signaling), CD44 (1:500—Abcam), β-actin (1:5000—Sigma), CD24 and VEGF (1:200 Santa Cruz). Standard western blotting procedures and analysis were performed following the method as described by Borin et al. [12].

### Protein antibody array

Proteins extracts was processed to evaluate the expression profiles from lung tissue by mouse cytokine antibody array (AAM-CYT-1000-8, RayBiotech, Inc.). 250 μg of protein sample was loaded to the membrane according to the manufacturer’s instructions, and the chemiluminescent reaction was detected using LAS-3000 imaging machine (Fuji Film, Japan). All of the emitted signals were analyzed using same region of interests (ROIs), and were normalized to the positive control, using Image J software.

### Flow cytometry analysis

Organs collected (n = 5 of each group) for flow analysis, were kept in 5mM PBS/EDTA, then disseminated into single cells, filtered through a 70 micron mesh, and spun at 1200 rpm for 15 minutes. The pellet was re-suspended in fresh 1x PBS Mg/Ca (Hyclone, GE Healthcare Life Sciences, Logan, Utah), and added mouse LEAF blocker (Biolegend) for 15 minutes. Next, cells were labeled to detected two distinct cell populations, endothelial and MDSCs. For endothelial population, CD31 (FITC), CD309 (PE), CD45 (PE/Cy5), CD133 (APC) markers were used, and for myeloid we used CD45 (FITC), CD11b (PE), Ly6C (PerCP), Ly6G (APC) markers. All antibodies were mouse specific and purchased from Biolegend. Flow cytometry samples were acquired using Accuri C6 flow cytometer (BD Biosciences), and analyzed by BD Accuri C6 software.

### Statistical analysis

Quantitative data were expressed as standard error of the mean (± SEM) and analyzed through *t Student* test or ANOVA (depending on the number of comparative groups) followed by Bonferroni test for normal distribution. Those groups with non-normal distribution was analyzed using the Mann-Whitney test or Kruskal-Wallis test. Statistical significant differences were considered at p value <0.05 using Graphpad prism 6 software.

## Results

### HPßCD-HET0016 decreases migration and invasion of breast cancer metastatic cells

**Fig 1:**
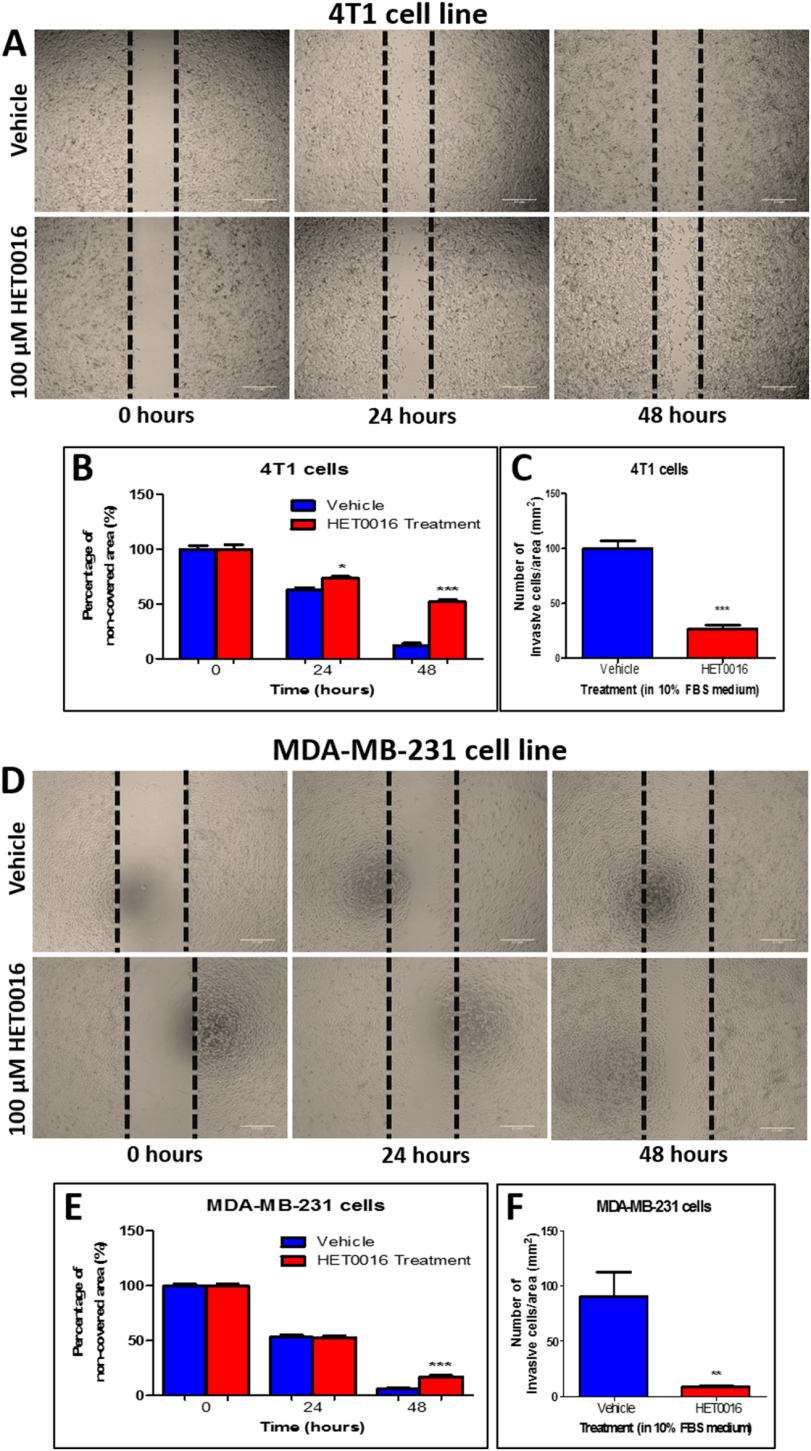
Cell migration and invasion assay carried out in 4T1 murine breast cancer cell line and MDA-MB-231 human breast cancer cell line after 24 and 48 hours of 100 μM HPßCD-HET0016 treatment. (A and D) Representative images of wound healing migration assay; (B and E) Semi-quantitative analysis of migration cells at non-covered area; (C and F) Semi-quantitative analysis of number of invasive cells per area after 48 hours of HPßCD-HET0016 treatment. Statistically significant differences, were verified by ANOVA followed by Bonferroni’s test. *p < 0.05 or ***p < 0.0001 in comparison to untreated cells (vehicle).

Triple negative metastatic breast cancer cell line MDA-MB-231 from human and 4T1 from mouse treated with vehicle or HPßCD-HET0016 were subjected to migration and invasion assay. After 24 hours of HPßCD-HET0016 treatment, during a wound healing migration assay, no significant difference was observed in the motility of both cell types compared to vehicle treatment. However, at 48 hours, HPßCD-HET0016 treated 4T1 cells showed a significant decrease in the motility (52 ± 1.65% less cell encroachment of the total area (wound)) compared to vehicle treated cells (12.2 ± 1.81% less cell encroachment of the total area; p < 0.0001 –Fig 1A and 1B). Likewise, MDA-MB-231 cells showed a similar reduction in cell migration after 48 hours of HPßCD-HET0016 treatment (17.3 ±1.26% of non-covered area) compared to 8.7% ± 1.39% of the non-covered area in control (Fig 1D and 1E).

HPßCD-HET0016 treatment was also able to decrease the invasion of both cell lines significantly, after 48 hours. In 4T1 cells, there was 73.4% (± 0.9%) of reduction in invasion compared to vehicle treated cells (p < 0.0001 - (Fig 1C). MDA-MB-231 cells followed the same pattern as of 4T1 cells about 77.8% (± 1.3%) of reduction compared to control vehicle treatment (Fig 1F).

### HPßCD-HET0016 reduces tumor volume and lung metastasis in an immunocompetent breast cancer mouse model

**Fig 2:**
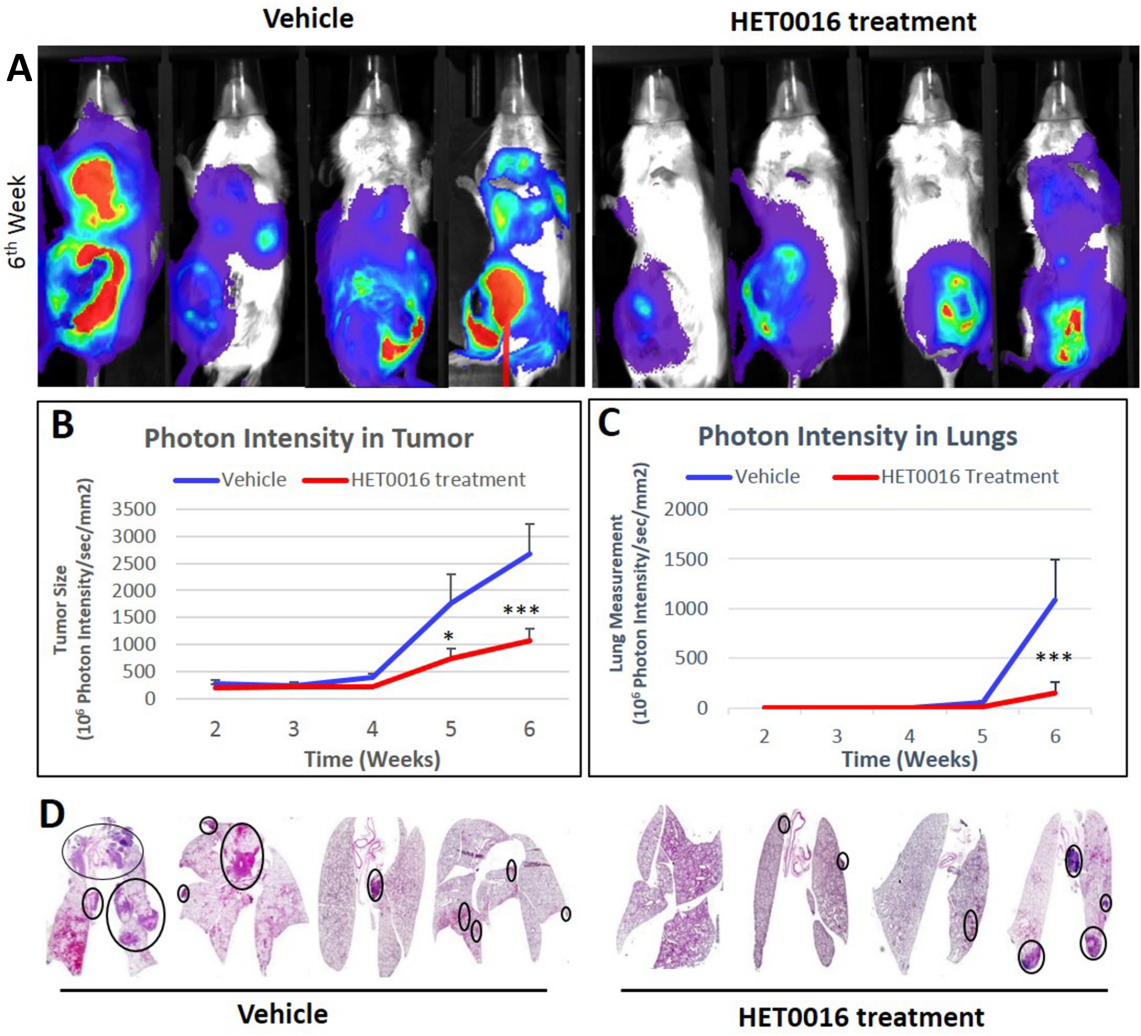
Tumor size and lung metastasis analysis of vehicle and HPßCD-HET0016 treatment groups acquired by optical imaging. (A) Representative images at 6^th^ week after cells inoculation into mammary fat pad; (B) Semi-quantitative analysis of the photon intensity in mammary fat pad area to determine tumor size; (C) Semi-quantitative analysis of the photon intensity in chest area to determine the presence of lung metastasis; (D) Histopathologic analyses by hematoxylin and eosin staining in lung tissues from vehicle and HET treatment groups. Images were taken 4x magnification. Circles indicate the sites of metastasis. Statistically significant differences, were verified by ANOVA followed by Bonferroni’s test. *p < 0.05 or ***p < 0.0001 in comparison to untreated group (vehicle) in the same time-point.

To observe whether HPßCD-HET0016 could decrease breast cancer metastasis, 4T1-luciferase positive cells were orthotopically implanted into mammary fat pad of female Balb/c mice. The animals were followed for 6 weeks and received three weeks of treatments starting on day 15 of tumor implantation. Optical images were obtained to verify the primary tumor growth and the presence of lung metastasis by measuring the photon activity from 4T1 luciferase positive cells. ROIs were drawn around the 4^th^ mammary fat pad (primary tumor) and lung (metastatic) areas to measure the photon intensity per sec per mm^2^ (Fig 2A). As expected, HPßCD-HET0016 treatment significantly decreased the photon intensity in primary tumor (p<0.001) and interestingly decrease the photon intensity in the lungs (p<0.0001) at 6 weeks of tumor implantation compared to vehicle treated animals (Fig 2B and 2C). H&E staining showed less foci of metastasis in lung of HPßCD-HET0016 treated animals compared to vehicle treated animals corroborating our optical imaging findings (Fig 2D). Besides, there were no statistical differences observed in body weights between vehicle and HPßCD-HET0016 treated groups throughout the study implying that the HPßCD-HET0016 treatment did not have any toxic effects on the animals.

### Reduction of metalloproteinases’ levels in the lungs of HPßCD-HET0016 treated animals via PI3K/AKT pathway

**Fig 3:**
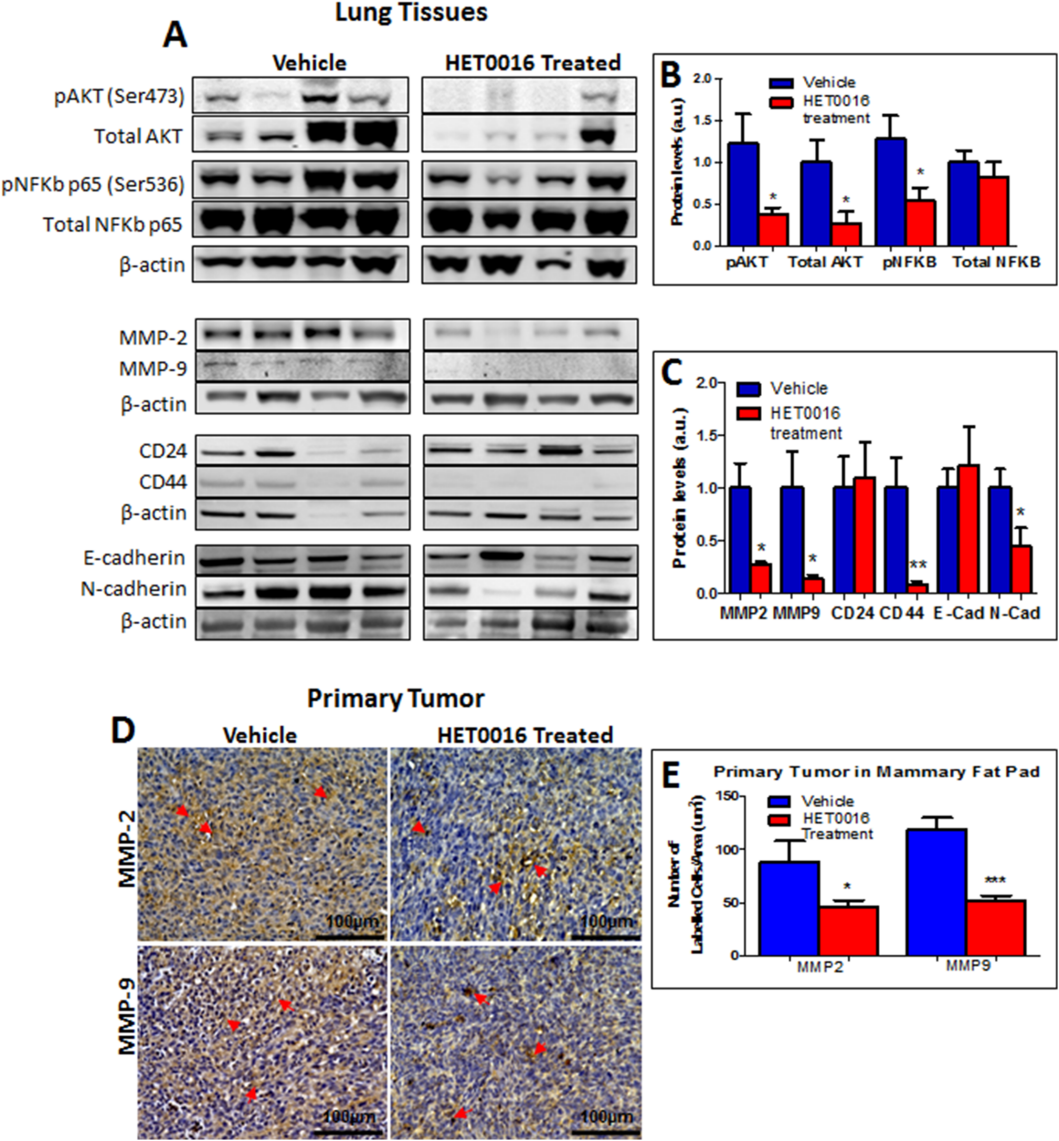
**Protein expression analyses in lung metastasis (A) and primary tumor (D)**. (A) Photomicrographs of Western blotting for pAKT, total AKT, pNFκB (p65 subunit), total NFκB (p65 subunit), MMP-2, MMP-9, CD24, CD44, E-cadherin, N-cadherin and β-actin in lungs tissues of vehicle and HPßCD-HET0016 treated group; (B) Semi-quantitative analysis (densitometry) of Western blotting for pAKT, total AKT, pNFκB (p65 subunit), total NFκB (p65 subunit) and (C) for MMP-2, MMP-9, CD24, CD44, E-cadherin, N-cadherin in lungs tissues of vehicle and HPßCD-HET0016 groups; (D) Representative pictures of immunostaining of primary tumor in vehicle and HPßCD-HET0016 treatment groups. (E) Semi-quantitative analysis of immunostaining for MMP-2, MMP-9 protein levels in primary tumor; Significant values from ANOVA followed by Bonferroni’s test are represented by mean ± SEM *p < 0.05 or ***p < 0.0001 in comparison to untreated group (vehicle). Images were taken 40x magnification. Arrows show the positive labelling.

To investigate the mechanism through which HPßCD-HET0016 treatment might induce the inhibition of metastatic processes, western blotting was performed toward two main pathways already identified to be activated by 20-HETE, namely; Mitogen-Activated Protein Kinase (MAPK) and PI3K pathways. The MAPK/ERK 1/2 pathway regulates VEGF and angiogenesis and the PI3K/AKT pathway regulates MMPs and invasion. Our results showed reduced protein levels of pAKT, total AKT, and pNFκB in lungs of animals treated with HPßCD-HET0016 compared to untreated animals (Fig 3A). Increased levels of pERK1/2 were found in the treated group, although, we observed no alterations in the levels of VEGF between those groups (supplementary data–Fig 1A). Cancer stem cell (CD44 and N-cadherin) and migration (MMP-2 and -9) markers showed significant decrease in the levels in treated group compared to control. However, cell adhesion markers such as CD24 and E-cadherin showed no significant differences between the vehicle and the treated groups (Fig 3A). Corroborating these findings, significantly reduced MMP-2 and -9 levels were also detected by IHC in primary tumors from mammary fat pad of animals treated with HPßCD-HET0016 (Fig 3B and 3C). CD44, CD24, E-cadherin and N-cadherin markers were evaluated as well. We found a tendency of increase in cell adhesion markers such as CD24 and E-cadherin and a decrease in stem cell markers such as CD44 and N-cadherin but no statistical difference was achieved (supplementary data–Fig 1B and 1C).

### HPßCD-HET0016 decreases expression of pro-inflammatory and growth factors and granulocytic MDSCs population in lung microenvironment

In order to assess whether HPßCD-HET0016 could influence the tumor microenvironment and decrease lung metastasis, we evaluated 40 cytokines, chemokines and growth factors related to proliferation, angiogenesis, inflammation, immunosuppression, migration/invasion, cell adhesion and chemotaxis as describe in Table 1, by protein antibody array using lung lysate from animals treated HPßCD-HET0016 or vehicle.

**Table 1:** Protein expression profile of 40 different cytokines, chemokines and pro-angiogenic factors in mouse lung metastasis.

Cytokines, Chemokines and Pro-angiogenic factors	P values	Vehicle		HPßCD-HET0016 treatment	
		Mean	SE	Mean	SE
ALK-1	p = 0.0980	12.43	1.872	24.47	9.201
bFGF	P = 0.4768	21.61	8.4	20.52	15.38
CXCL16	Not detected				
E-Cadherin	P = 0.2167	233.9	33.28	176.4	57.06
**EGF**	P = 0.0190* **↓**	**56.56**	**5.759**	**17.26**	**10.99**
Endoglin	P = 0.3717	81.12	9.943	95.96	41.12
Eotaxin	P = 0.4628	8.724	1.177	7.543	11.84
**E-Selectin**	P = 0.0287* **↑**	**28.09**	**3.346**	**42.67**	**2.954**
**Fas**	P = 0.0475* **↓**	**40.65**	**13.86**	**9.716**	**3.070**
GM-CSF	P = 0.0934	78.66	3.696	59.21	11.65
HGF	P = 0.2551	9.8	1.545	3.676	8.339
HGFR	P = 0.3146	53.57	6.867	45.17	14.55
ICAM-1	P = 0.2304	152.4	25.42	197.9	49.61
IFN-γ	P = 0.4515	29.99	5.439	28.07	13.79
IGFBP-2	P = 0.4579	68.16	9.764	65.28	23.61
IGF-I	P = 0.2051	71.3	19.52	46.13	19.23
IGF-II	P = 0.3698	65.74	6.069	57.94	21.03
IL-10	P = 0.4177	53.12	8.654	49.12	15.46
IL-12p40/p70	P = 0.4269	97.37	19.15	104.0	27.85
IL-13	P = 0.0602	39.0	8.673	60.08	3.966
**IL-17A**	P = 0.0150* **↓**	**70.89**	**2.434**	**25.15**	**8.983**
**IL-1 beta**	P = 0.0227* **↓**	**31.35**	**3.226**	**15.25**	**4.581**
**IL-4**	P = 0.0441* **↓**	**55.83**	**6.252**	**36.9**	**5.658**
IL-6	P = 0.2970	45.72	3.009	40.14	9.168
IL-6R	P = 0.4155	22.56	2.694	19.87	11.50
KC	P = 0.3918	38.06	5.418	34.16	12.11
MDC	P = 0.4830	42.64	9.894	41.80	15.76
MCP-1	P = 0.2413	50.44	10.29	37.48	13.23
MIP-1 alpha	P = 0.3496	28.91	2.561	25.30	8.303
**MMP-2**	P = 0.0519* **↓**	**38.86**	**10.05**	**9.943**	**1.285**
RANTES	P = 0.3646	115.5	24.64	126.9	18.27
**SCF**	P = 0.0220* **↑**	**12.6**	**5.847**	**30.72**	**2.196**
**SDF-1 alpha**	P = 0.0374* **↓**	**27.10**	**8.751**	**-5984**	**8.109**
TIMP-2	P = 0.0910	12.96	8.691	-7.081	7.431
TNF alpha	P = 0.3471	61.44	13.23	55.01	7.486
VEGF-A	P = 0.1060	8.614	3.335	30.85	14.61
VEGFR1	P = 0.1413	33.53	12.95	59.47	16.41
VEGFR2	P = 0.3733	70.65	11.76	64.57	13.03
VEGFR3	P = 0.2911	3.644	1.608	5.468	2.297
VEGF-D	P = 0.1193	15.46	2.455	10.76	2.350

ALK-1: Activin receptor-like kinase-1; bFGF: Fibroblast growth factor (basic); CXCL16: Chemokine (C-X-C motif) ligand 16; EGF: Epidermal growth factor; Fas: Fas ligand or CD95 ligand; GM-CSF: Granulocyte-macrophage colony stimulating factor; HGF: Hepatocyte growth factor; HGFR: Hepatocyte growth factor receptor; ICAM-1: Intercellular adhesion molecule 1; IFN-γ: Interferon gamma; IGFBP-2: Insulin-like growth factor-binding protein 2; IGF-I: insulin-like growth factor 1; IGF-II: insulin-like growth factor 2; IL-10: Interleukin-10; IL-12p40/p70: Interleukin-12 subunit p40 and p70; IL-13: Interleukin-13; IL-17A: Interleukin-17A; IL-1β: Interleukin-1 beta; IL-4: Interleukin-4; IL-6: Interleukin-6; IL-6R: Interleukin-6 receptor; KC: Chemokine (C-X-C motif) ligand 1 (CXCL1); MDC: Macrophage-derived chemokine or Chemokine (C-C motif) ligand 22 (CCL22); MCP-1: Monocyte chemoattractant protein-1; MIP-1α: Macrophage inflammatory protein 1-alpha or Chemokine (C-C motif) ligand 3 (CCL3); MMP-2: Matrix metalloproteinase 2; RANTES: Regulated on activation, normal T cell expressed and secreted or Chemokine (C-C motif) ligand 5 (CCL5); SCF: Stem cell factor; SDF-1α: Stromal cell-derived factor 1 alpha; TIMP-2: Tissue inhibitor of metalloproteinases-2; TNFα: Tumor necrosis factor alpha; VEGF-A: Vascular endothelial growth factor A; VEGF-R1: Vascular endothelial growth factor receptor 1; VEGF-R2: Vascular endothelial growth factor receptor 2; VEGF-R3: Vascular endothelial growth factor receptor 3; VEGF-D: Vascular endothelial growth factor D. All antibodies are prepared in duplicate.

*: significant P value <0.05.

**Fig 4:**
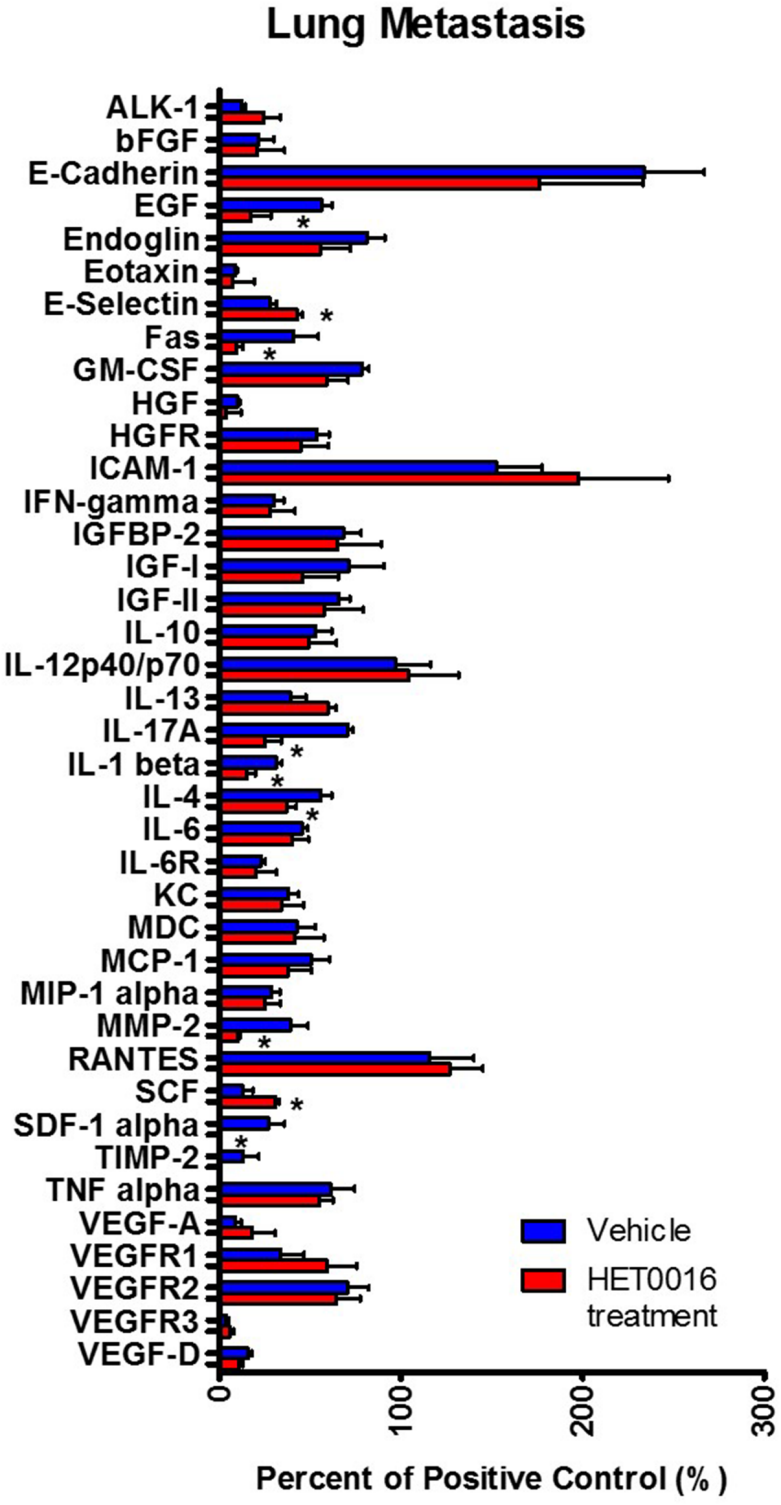
Protein expression profile was performed using a custom designed membrane-based protein array. Quantitative estimation of pro-angiogenic growth factors, chemokines, and cytokines in lungs tissue from vehicle and HPßCD-HET0016 treated groups; Significant values from t student test followed by Mann-Whitney’s test are represented by mean ± SEM *p < 0.05 in comparison to untreated group (vehicle).

HPßCD-HET0016 was able to significantly decrease the protein expression of EGF, FasR, SDF-1α, IL-1β, IL-4, IL-17A, MMP-2 and increase the expression of SCF and E-selectin in lungs of animals treated with HPßCD-HET0016 compared to vehicle treated animals (Fig 4).

**Fig 5:**
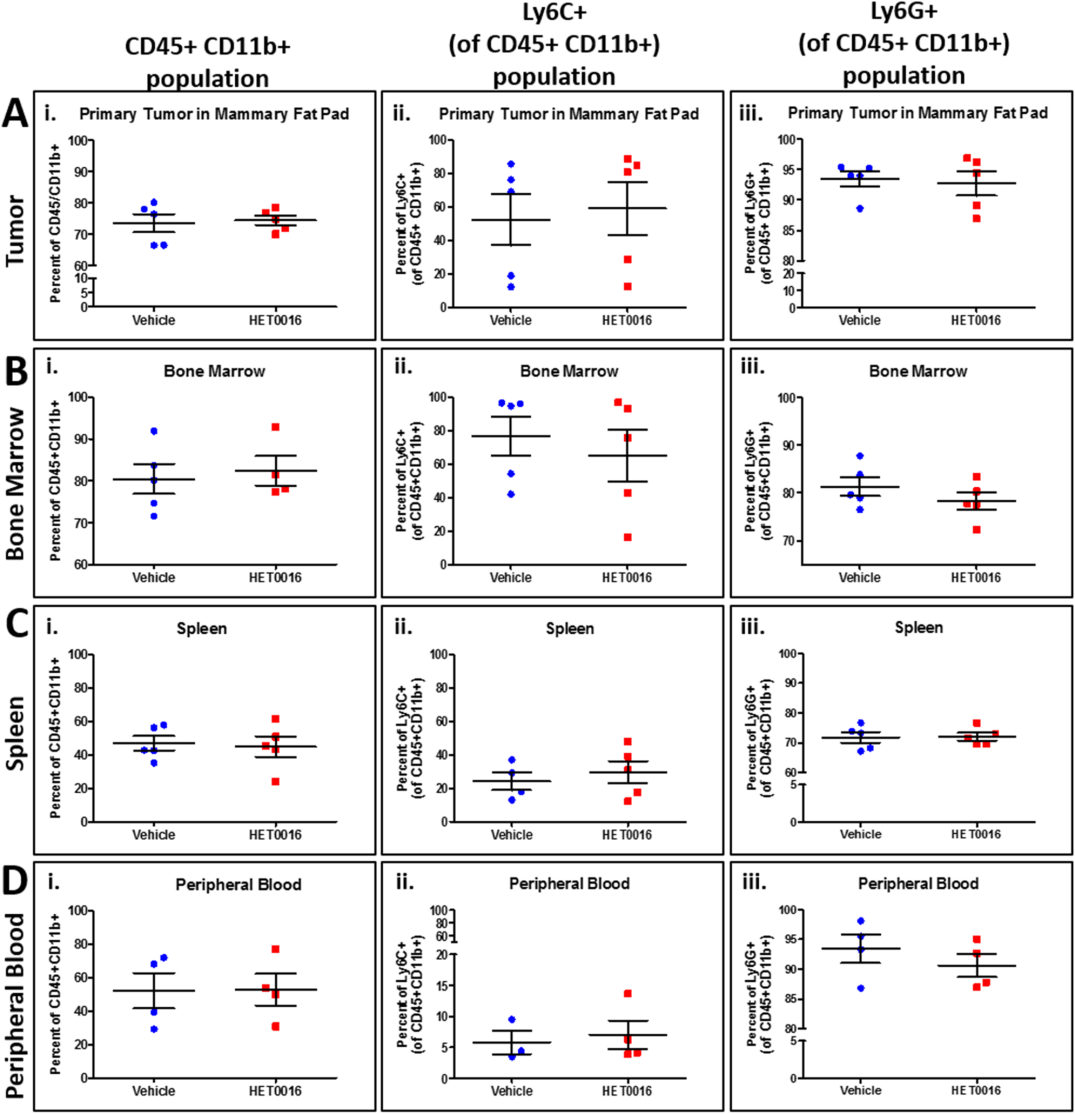
MDSCs evaluation in vehicle and HPßCD-HET0016 treated groups. **(**A) Primary tumor in mammary fat pad; (B) Bone marrow tissue; (C) Spleen and (D) Peripheral blood samples. It was used mouse specific antibodies for CD45, CD11b, Ly6C, Ly6G markers detected by Accuri C6 flow cytometer (BD Biosciences). The samples values were submitted to *t student* test followed by Mann-Whitney’s test, however no statistical difference were found between vehicle and treated groups.

**Fig 6:**
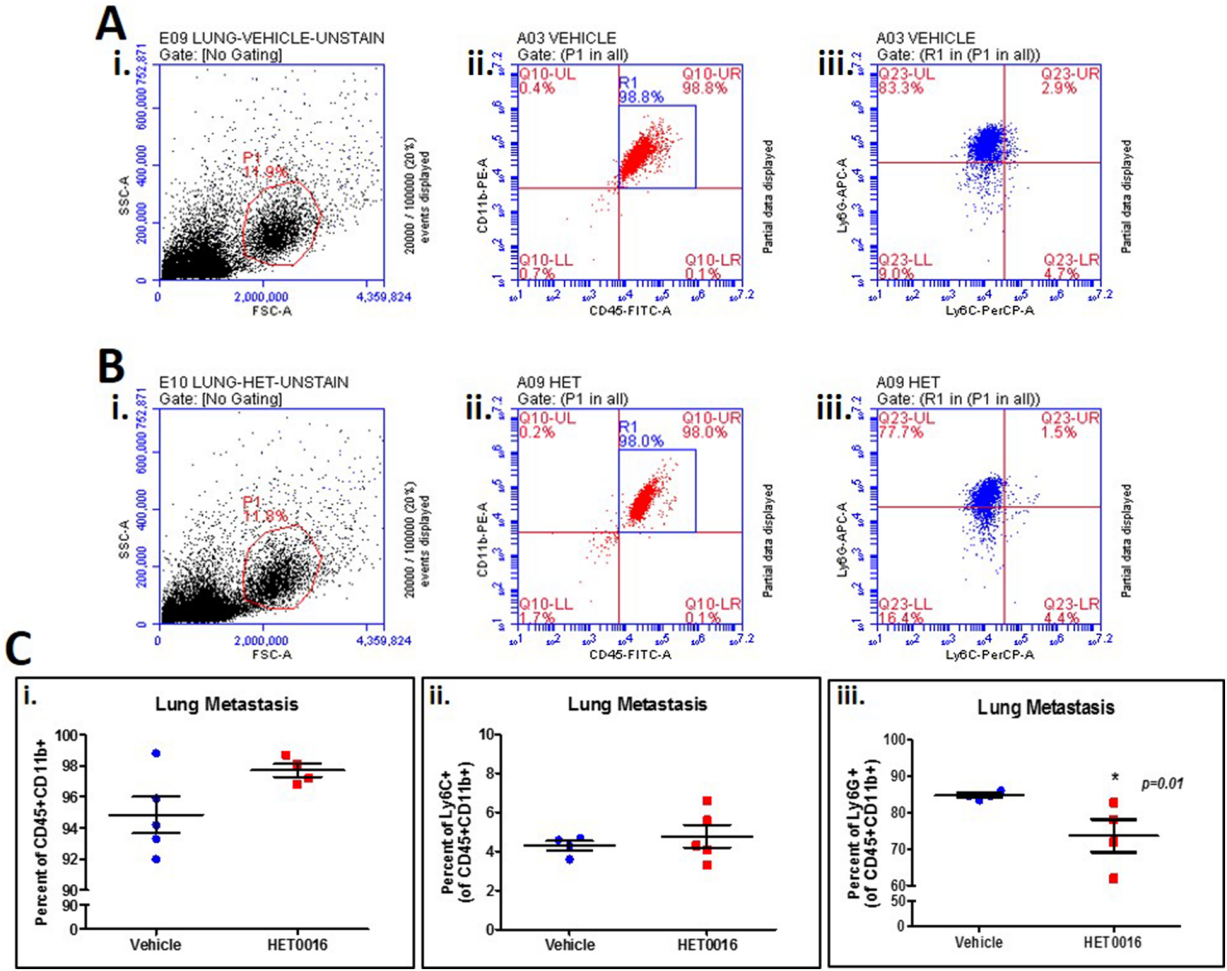
MDSCs identification in lung metastatic tissue after vehicle or HPßCD-HET0016 treatment. Representative graphs of (A) vehicle group sample and (B) HPßCD-HET0016 treated group, gated in: (i) Forward scatter (FSC) vs. side scatter (SSC) area to delimitate alive cells population; (ii) Identification of CD45+ and CD11b+ positive cells in R1; (iii) Quantification of granulocytic and monocytic MDSCs population by Ly6G+ and Ly6C+ separation. (C) Quantitative analyses of MDSCs population in lung metastatic samples of vehicle and HPßCD-HET0016 treated group, showing a reduction of G-MDSCs in lungs samples treated with HPßCD-HET0016. Significant values from *t student* test followed by Mann-Whitney’s test are represented by mean ± SEM *p < 0.05 in comparison to vehicle group.

Furthermore, blood, spleen, lungs, bone marrow, and tumor samples from treated and vehicle groups were collected for flow cytometer analysis to identify changes in the phenotypes of myeloid and endothelial cell populations. However, no changes in myeloid or endothelial cell phenotypes were observed between vehicle and HPßCD-HET0016 treated animals in cells collected from blood, spleen, bone marrow and tumor (Fig 5A, 5B, 5C and 5D). On the other hand, cells collected from lungs showed significant decrease (p<0.01) in the granulocytic MDSCs (CD45+ CD11b+ Ly6G+) population (Fig 6A, 6B and 6C), confirming the importance of HPßCD-HET0016 in inhibiting metastases by decreasing the recruitment of MDSCs. Even though we observed no significant differences in the CD11b+ myeloid cells infiltration in both HPßCD-HET0016 treated and untreated groups, surprisingly the Ly6G+ cells gated on CD11b+ cells decreased significantly in the HPßCD-HET0016 treated group. This led us to believe that, while the availability of CD11b+ myeloid cells remains unchanged in both groups, HPßCD-HET0016 impedes the polarization of the available CD11b+ myeloid cells to Ly6G+ MDSC phenotype (G-MDSCs), thereby decreasing overall immunosuppressive environment in TME.

## Discussion

In our previous studies, we observed the effect of blocking the 20-HETE pathway with HPßCD-HET0016, a highly selective inhibitor, which involves the CYP4A and CYP4F families in inhibiting tumor angiogenesis, proliferation, migration, and regulation, in human breast cancer and glioma models [8,9]. Once 20-HETE is blocked by HPßCD-HET0016, there are at least two possible signaling pathways inactivated, which can regulate angiogenesis and invasion. One of them, PI3K/AKT pathway in the present study was attenuated by HPßCD-HET0016 treatment, leading to a reduction in MMP-2 and -9 levels and also the migration and invasion of tumor cells consequently reducing lung metastasis (supplementary data–Fig 2). On the other hand, MAPK/ERK1/2 pathway activation was increased after treatment which could have potentially annulled the effects of our treatment. However, no alterations in the VEGF levels between treated and control groups were found. Overall, ERK1/2 activation mediates transcription factors to regulate gene expression conferring a survival advantage to cells. Nonetheless, studies have demonstrated that persistent activation of ERK also contributes to cell death [13], differentiation [14] or might be related to the mechanisms of drug resistance [15]. Our study corroborates with other studies, which have shown that 20-HETE inhibition reduces invasion in vitro, and angiogenesis and metastasis in vivo by decreasing VEGF and MMP-9 expression in human non-small cell lung cancer and MDA-MB-231 breast cancer mouse models [16,17].

P450-derived eicosanoids overexpression can promote human cancer metastasis by increasing CD44 expression [18] and our results demonstrated a significant decrease in cancer stem cell markers, such as CD44 and N-cadherin in lungs of HPßCD-HET0016 treated group and in the primary tumor. However, cell adhesion markers such as CD24 and E-cadherin showed no significant differences between the vehicle and the treated groups. In breast cancer, a significant increase in CD44 and its variants has been found in malignant and premalignant lesions compared to normal breast tissue providing further confirmatory evidence to the role of CD44 in the development of distal metastasis and tumor progression [19].

Attention also has shifted to CD24 due to its important role in the development of cancer metastases and as a marker of malignancy in several tumor types. In fact, a subpopulation of cancer cells enriched as tumor-initiating cells with the antigenic phenotype CD44+/CD24- has been increasingly found in metastatic breast cancer, in comparison to the majority of carcinoma cells, which are CD44-/CD24+ phenotype [20–23]. The ability of a single cell to move from the primary tumor to metastatic site is facilitated through the transition from an epithelial phenotype (CD44+/CD24+) to a mesenchymal phenotype (CD44+/CD24-) [21,23]. However, a meta-analysis of sixteen studies comprising of 5,697 breast cancer cases showed, recently, that CD24 overexpression was significantly associated with shortened overall survival, while no such association was observed between CD44 and CD44+/CD24- phenotypes, suggesting a large-scale study is necessary to confirm these findings [24].

We demonstrated that HPßCD-HET0016 was able to significantly decrease the protein expression of EGF, Fas, and SDF-1α (a strongly chemoattractant for CXCR4+ lymphocytes and stem cells). FasR/Fas ligand (CD95) is one of the most important apoptotic pathway used by immune cells to avoid cancer development. Interestingly, Fas ligand, a type-II transmembrane protein that belongs to the tumor necrosis factor (TNF) family, usually found bound to the cell membrane, can also be released by metalloprotease cleavage [25,26]. Evidence suggests that Fas ligand preferentially induces apoptosis, however, its role is also related to a greater degree to the induction of cell migration [26]. The decrease of Fas levels found in our study might also suggest a reduction in migration and invasion of tumor cells and the granulocytes population, in addition to the reduced levels of MMP-2 and -9. We also report here that HPßCD-HET0016 can reduce pro-inflammatory interleukins such as IL-1β, IL-4 and IL-17A, and granulocytes (CD45+ CD11b+ Ly6G+) population in lungs of treated group.

It is known that the dynamic network of cytokines, chemokines, growth and inflammatory factors, alter the local balance of tumor microenvironment during cancer development. IL-1β, a mediator of pro-inflammatory response is also produced by metastatic breast cancer cells (MDA-MB-231 and MDA-MB-436) [27]. IL-1β indirectly induces motility in cancer cells through mesenchymal stem cells (MSC)-dependent release of chemokines such as CXCL1, 2, 3, 5, 6, 8, CCL2, 3, 5, and 20 suggesting correlation between interaction of these cells and the metastatic potential of the breast cancer cells [27]. Moreover, IL-4 interferes with the apoptotic program activated by anticancer agents in tumor cells. Conticello et al. [28] reported that IL-4 stimulation resulted in up-regulation of anti-apoptotic protein as cFLIP/FLAME-1 and Bcl-xL, inhibiting apoptosis induced by CD95 (Fas/APO-1) and chemotherapeutic drug stimulation in primary prostate, breast, and bladder cancer. Primary breast cancer cells acquired sensitivity to apoptosis in vitro only in the absence of IL-4 [28].

Besides that, IL-17A has been considered as a potent activator of pro-tumor innate immunity by regulation and suppression of T lymphocyte function in the tumor microenvironment [29]. In breast tumors, IL-17A is associated with ER negative or triple negative tumors and poor prognosis. IL-17A can promote proliferation, stimulate cell migration and invasion and resistance to conventional chemotherapy [30]. According to Chung et al. [31], IL-17A mediates paracrine network to promote tumor resistance to anti-angiogenic therapy by inducing the expression of GM-CSF (through NFκB and ERK signaling) to mobilize and recruit MDSCs to the tumor microenvironment. In our data, GM-CSF showed a tendency of decrease after HPßCD-HET0016 treatment in lungs (Table 1, line 11).

Our data also shows an increased expression of SCF and E-selectin in treated group. It has been shown that E-selectin assists shear-resistant adhesion of circulating tumor cells to the vessel surface under dynamic blood flow [32]. Furthermore, SCF is a potent chemokine that favors recruitment of cancer-stem cells and other inflammatory cells including MDSCs [33,34]. In the light of these evidences, our data shows an increased levels of these proteins which could be related to the relapse and refractoriness of the tumor showing resistance to our treatment. Also, the increase of these proteins might be a transient compensatory mechanism adopted by the tumor to overcome the therapeutic insult but are not be the sole factors driving metastasis in the context of HPßCD-HET0016 treatment.

In fact, the tumor microenvironment secretes many factors such as GM-CSF, VEGF, SCF, IL-6, IL-10, IL1β, PGE2, cyclooxygenase enzyme (COX) and the complement component C5a which promote recruitment and accumulation of immature MDSCs [35]. We have decided to look at MDSCs in lung metastasis as these cells have been associated to the creation of pre-metastatic lung niches by producing large amounts of MMP-9 and proinflammatory cytokines promoting vascular remodeling [36]. We, likewise, have seen the same in our study.

MDSCs are a heterogeneous population of myeloid cells composed of myeloid progenitor cells and immature myeloid cells which are commonly defined by the markers CD11b (αM-integrin) and Gr-1, wherein Gr-1 constitutes two subsets which can also be identified by Ly6C marker for monocytes (M-MDSCs) and Ly6G marker for granulocytes (G-MDSCs) [37,38]. G-MDSCs are the majority of all MDSCs representing more than 75% of the population in cancer patients and tumor-bearing mice [38]. Wculek and Malanchi [39] found CD11b+ Ly6G+ cells accumulated in the lungs before cancer cells infiltrated the tissue, increasing their number after metastatic colonization. The soluble factors released by G-MDSCs are able to suppress T-cell responses [38,40]. In contrast, when mice bearing liver metastasis in breast cancer model were treated with purified anti-Ly6G rat monoclonal antibody, the Ly6G+ cells were depleted by about 90% of the total peripheral blood cells which was able to reduce hepatic metastasis [41].

Furthermore, G-MDSCs have been considered an important pre-requisite in the general process of angiogenesis, tumor progression and nowadays in metastasis. Our results indicate the importance of distal metastatic microenvironment in the creation of a niche for future metastasis and decreased level of G-MDSCs in the lungs following HPßCD-HET0016 treatment indicates the potential of 20-HETE synthesis blocker to inhibit breast cancer metastasis. On this account, therapeutic strategies to decrease the G-MDSCs population show promise in preventing metastasis. It will be worthwhile to direct the current efforts toward the elucidation of the role of T-cell response in the context of this treatment.

## Conclusions

In summary, our data demonstrated that HPßCD-HET0016 decreases MMP-2 and -9, CD44, and N-cadherin expression, migration and invasion of tumor cells and consequently reduces lung metastasis. HPßCD-HET0016 treatment was also able to significantly decrease the protein expression of EGF, Fas, and SDF-1α in conjunction with the reduced levels of MMP-2 and -9, and the granulocytic MDSC population result in an overall attenuation of migratory and invasive properties of cancer cells.

We are also the first group to report that HPßCD-HET0016 can reduce pro-inflammatory interleukins such as IL-1β, IL-4 and IL-17A and granulocytic (CD45+ CD11b+ Ly6G+) populations in the lungs of treated animals’ group. Taking a step forward in this direction, we are currently working on establishing the role of tumor-derived exosomes in the initiation, formation and development of metastatic niche in distant organs. We seek to identify the tumor-derived exosomal factors that are crucial in priming distant organs and thereby serving to create an immunosuppressive pre-metastatic niche for the invading tumor cells. We are looking towards employing HPßCD-HET0016 as a novel therapeutic drug for potentially blocking the tumor-derived exosomes in creating a niche for development of metastasis in distant organs.

## Supporting information

S1 Fig **Protein expression analyses in lung metastasis (A) and primary tumor (B).** (A) Photomicrographs of Western blotting detection for pERK, total ERK, VEGF and β-actin in lungs tissues of vehicle and HPßCD-HET0016 treated group. Increased levels of pERK1/2 were found in the treated group, although, no alterations in VEGF levels was found between those groups. (B) Semi-quantitative analysis (densitometry) of Western blotting for pERK, total ERK and VEGF in lungs tissues of vehicle and HPßCD-HET0016 groups; (C) Immunohistochemistry semi-quantitation for CD24, CD44, E-cadherin and N-cadherin in primary tumor showing a tendency of increase in cell adhesion markers such as CD24 and E-cadherin and a decrease in stem cell markers such as CD44 and N-cadherin. No significant statistical value was achieved; (D) Representative pictures of immunostaining of primary tumor in vehicle and HPßCD-HET0016 treatment groups. Images were taken 40x magnification. Arrows show the positive labelling.
(TIF)

S2 Fig Signaling pathway analysis showing (in)activation of angiogenesis, chemotaxis and inflammation process via NFkB/p65 and PI3K/AKT. The Ingenuity Pathway Analysis software (IPA—Qiagen) was used to determine the molecular networks. Data presented in Fig 3 suggests that AKT-mediated PI3 kinase pathway and p65 mediated canonical NFkB signaling pathway are significantly inhibited in the metastatic lung following the HPßCD-HET0016 treatment. The cytokines profile reported in our manuscript (Fig 4) contain important factors related to chemotaxis or recruitment activity which correspond to the activation of MDSCs recruitment.
(TIF)

## Abbreviations

AKT: Protein kinase B
Bcl-xL: B-cell lymphoma extra-large transmembrane protein
CD95: Fas/APO-1 receptor
cFLIP/FLAME-1: Caspase-8 and FADD-like apoptosis regulator
COX: Cyclooxygenase enzyme
DAB: Diaminobenzidine tetrachloride
DMEM: Dulbecco’s modified Eagle’s medium
EGFR: Epidermal growth factor receptor
ERK: Extracellular signal–regulated kinases
Fas: Fas ligand or CD95 ligand
FBS: Fetal bovine serum
GM-CSF: Granulocyte-macrophage colony stimulating factor
HPßCD: 2-Hydroxypropyl beta cyclodextrin
20-HETE: 20-Hydroxyeicosatetraenoic acid
IL: Interleukin
MDA-MB-231 or MDA-MB-436: Triple negative metastatic human breast cancer cell line
MDSCs: Myeloid-derived suppressive cells
MMPs: Matrix metalloproteinases
NFκB: Nuclear factor kappa-light-chain-enhancer of activated B cells
PET: Polyethylene terephthalate
PGE2: Prostaglandin E2
PI3K: Phosphatidylinositol-3-kinases
ROIs: Region of interests
RPMI: Roswell Park Memorial Institute 1640 medium
SCF: Stem cell factor
SDF-1α: Stromal cell-derived factor 1 alpha
SEM: Standard error of the mean
MAPK: Mitogen-Activated Protein Kinase
4T1: Triple negative metastatic murine breast cancer cell line
TME: Tumor microenvironment
TNFα: Tumor necrosis factor alpha
