# Supplementary figures and images for: HET0016 decreases lung metastasis from breast cancer in immune-competent mouse model
Source: PLoS One. 2017 Jun 13;12(6):e0178830. doi: 10.1371/journal.pone.0178830 (PMC5469456; doi:10.1371/journal.pone.0178830)

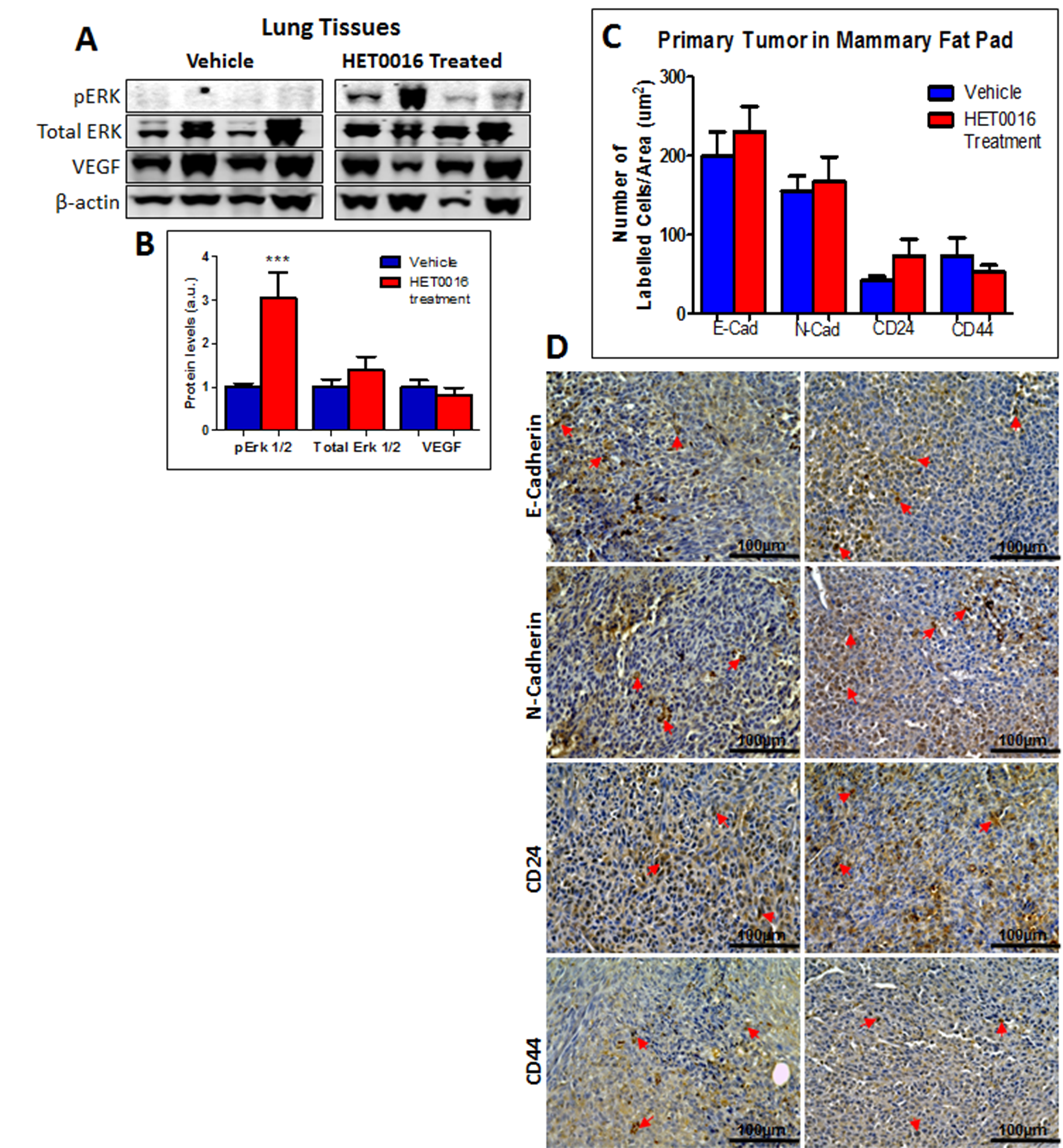

Supplement: S1 Fig — Protein expression analyses in lung metastasis (A) and primary tumor (B). (A) Photomicrographs of Western blotting detection for pERK, total ERK, VEGF and β-actin in lungs tissues of vehicle and HPßCD-HET0016 treated group. Increased levels of pERK1/2 were found in the treated group, although, no alterations in VEGF levels was found between those groups. (B) Semi-quantitative analysis (densitometry) of Western blotting for pERK, total ERK and VEGF in lungs tissues of vehicle and HPßCD-HET0016 groups; (C) Immunohistochemistry semi-quantitation for CD24, CD44, E-cadherin and N-cadherin in primary tumor showing a tendency of increase in cell adhesion markers such as CD24 and E-cadherin and a decrease in stem cell markers such as CD44 and N-cadherin. No significant statistical value was achieved; (D) Representative pictures of immunostaining of primary tumor in vehicle and HPßCD-HET0016 treatment groups. Images were taken 40x magnification. Arrows show the positive labelling. (TIF) [file pone.0178830.s001.tif]

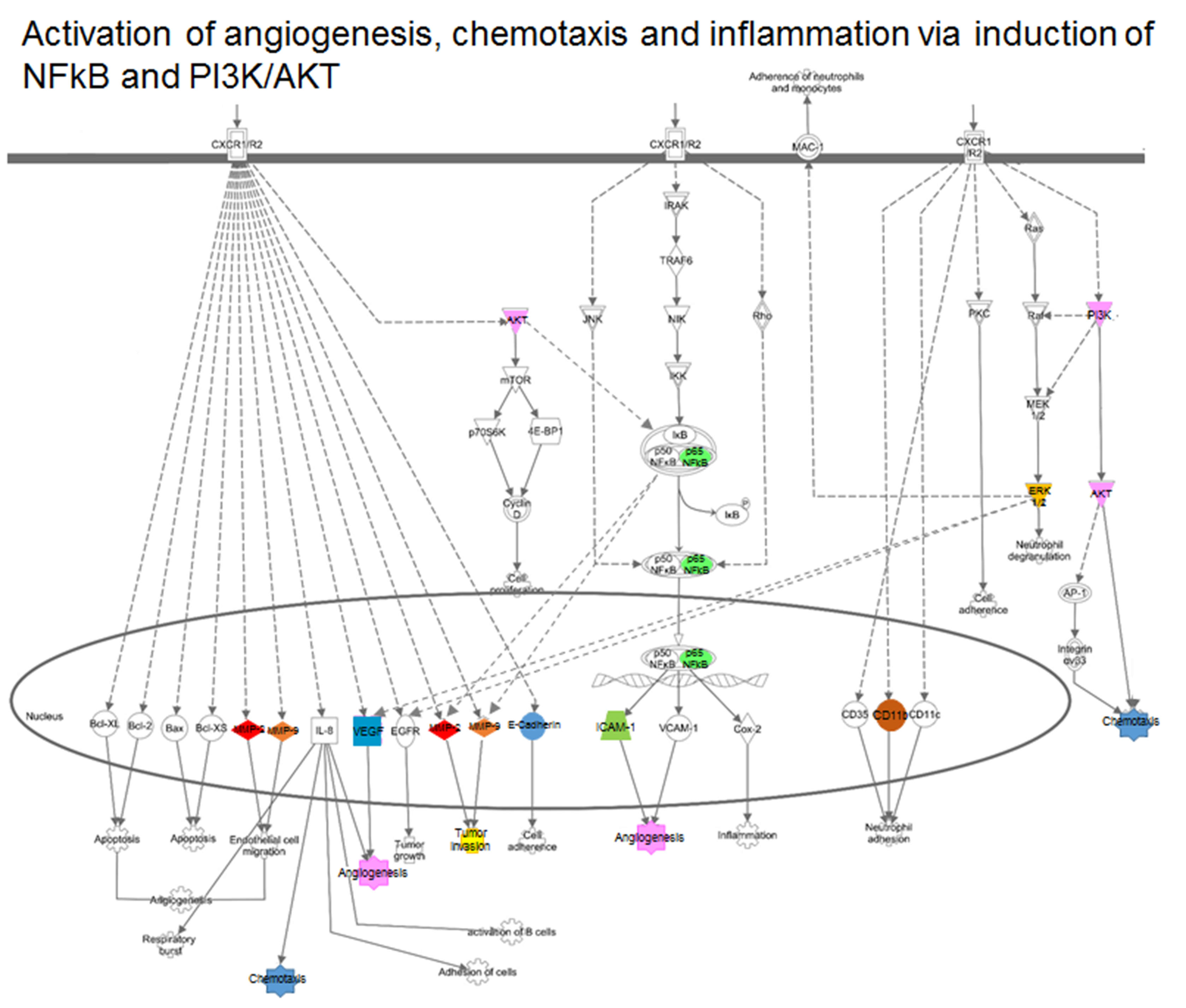

Supplement: S2 Fig — The Ingenuity Pathway Analysis software (IPA—Qiagen) was used to determine the molecular networks. Data presented in Fig 3 suggests that AKT-mediated PI3 kinase pathway and p65 mediated canonical NFkB signaling pathway are significantly inhibited in the metastatic lung following the HPßCD-HET0016 treatment. The cytokines profile reported in our manuscript (Fig 4) contain important factors related to chemotaxis or recruitment activity which correspond to the activation of MDSCs recruitment. (TIF) [file pone.0178830.s002.tif]
